# Exercise performance at different phases of the menstrual cycle: measurements, differences, and mechanisms - a narrative review

**DOI:** 10.3389/fendo.2025.1448686

**Published:** 2025-12-16

**Authors:** Yiqing Wen, Binghong Gao, Renwei Wang, Can Zhao

**Affiliations:** 1 College of Athletic Performance, Shanghai University of Sport, Shanghai, China; 2 School of Exercise and Health, Shanghai University of Sport, Shanghai, China

**Keywords:** athletes, eumenorrheic women, exercise performance, menstrual cycle phase, women

## Abstract

With the increasing participation of women in sports, research on women’s sports has gradually increased but remains less than that on men’s sports as a whole. The menstrual cycle is a unique physiological feature of women. Hormone fluctuations during this period can have a potential effect on exercise performance through various mechanisms, including substrate metabolism, cardiopulmonary function, body temperature regulation, and psychological factors. This narrative review analyzed the changes in aerobic performance, anaerobic performance, and strength during the menstrual cycle by analyzing domestic and international research on exercise performance at different phases of menstrual cycle and preliminarily discussed the influence of menstrual cycle-related factors on exercise performances. Results show that although a considerable proportion of female athletes believe that their exercise performance is affected by their menstrual cycle (poor exercise performance during the early follicular and midluteal phases), various exercise performance during different phases of the menstrual cycle are inconsistent. Such variability may be related to the inconsistency of current research methods, such as the method for identifying menstrual cycle phases. Current research focuses on aerobic, anaerobic, and strength indices, and only a few studies on speed, sensitivity, and flexibility exist. Studies that support the differences in transport performance during different menstrual cycle phases have the overall consensus that aerobic performance in the follicular phase is higher than that in the luteal phase and the effect of hormonal fluctuations on aerobic performance during the menstrual cycle can be reduced by ingesting glucose. Maximum strength is poorest during the luteal phase. However, strength training can be planned on the basis of the hormone fluctuation characteristics of the menstrual cycle. Most studies have shown that anaerobic performance is unaffected by the menstrual cycle. Further research is needed to quantify exercise performance during different phases of the menstrual cycle and determine the relevant factors affecting exercise performance to understand women’s exercise performance fully. It provides help to athletes in regulating their physiological cycle before games and ensuring their maximum performance during competition.

## 1 Introduction

The influence of female athletes on international competitive sports is increasing daily. The proportions of participants in previous Olympic Games (2.2% in the first session to 48.7% in the 32nd session) and participating projects (2.1% in the first session to 46% in the 32nd session) are constantly rising (1). The contribution of female athletes to China’s competitive sports is also growing. The proportion of female athletes in China who won gold exceeded that of men from the 24th to the 32nd Olympic Games and that in the 32nd Olympic Games reached 64%. China’s mass women’s sports participation has gradually increased, and women’s fitness enthusiasm has been significantly higher than that of male (2).

From puberty to perimenopause, women experience fluctuations in ovarian hormones due to menstruation. Estrogen and progesterone can fluctuate by 5 and 50 times, respectively (3). Studies have shown that hormones are an important factor affecting the development of exercise performance: estrogen and progesterone can not only affect nerves and muscles (4), they also affect substrate metabolism, ventilation, immunity, cognition, body temperature, the cardiovascular system, and other aspects (5–7). A close relationship exists between women’s exercise performance and the menstrual cycle (MC). Although women’s sports participation rate and performance are increasing, the research on men’s sports continues to predominate and that on women’s sports is scarce (8). Differences exist between men and women in terms of bone morphology, biomechanics, reproductive and endocrine systems, and other aspects. The results of male exercise research are not fully applicable to women (3). In addition, the specific problems associated with women’s menstruation remain to be solved. Such problems include the potential effects of exercise-induced MC disorders, changes in the MC through drugs, and coincidence of major competitions with the LF phase. Given that the MC is an unavoidable physiological law of the life processes of women, especially those who are athletes, and affects sports participation and performance, balancing the relationship between the MC and exercise performance remains a problem that has not been overcome in the field of competitive sports. In light of this context, the present study synthesizes and categorizes relevant domestic and international research on exercise performance across different phases of the MC. Building on these reviewed literatures, it further analyzes the relationship between the MC and exercise performance, aiming to provide a foundational reference for the manual regulation of the MC and to support the optimal utilization of exercise performance in major competitions.

### 1.1 Characteristics and rules of different phases of the MC

**Figure 1:**
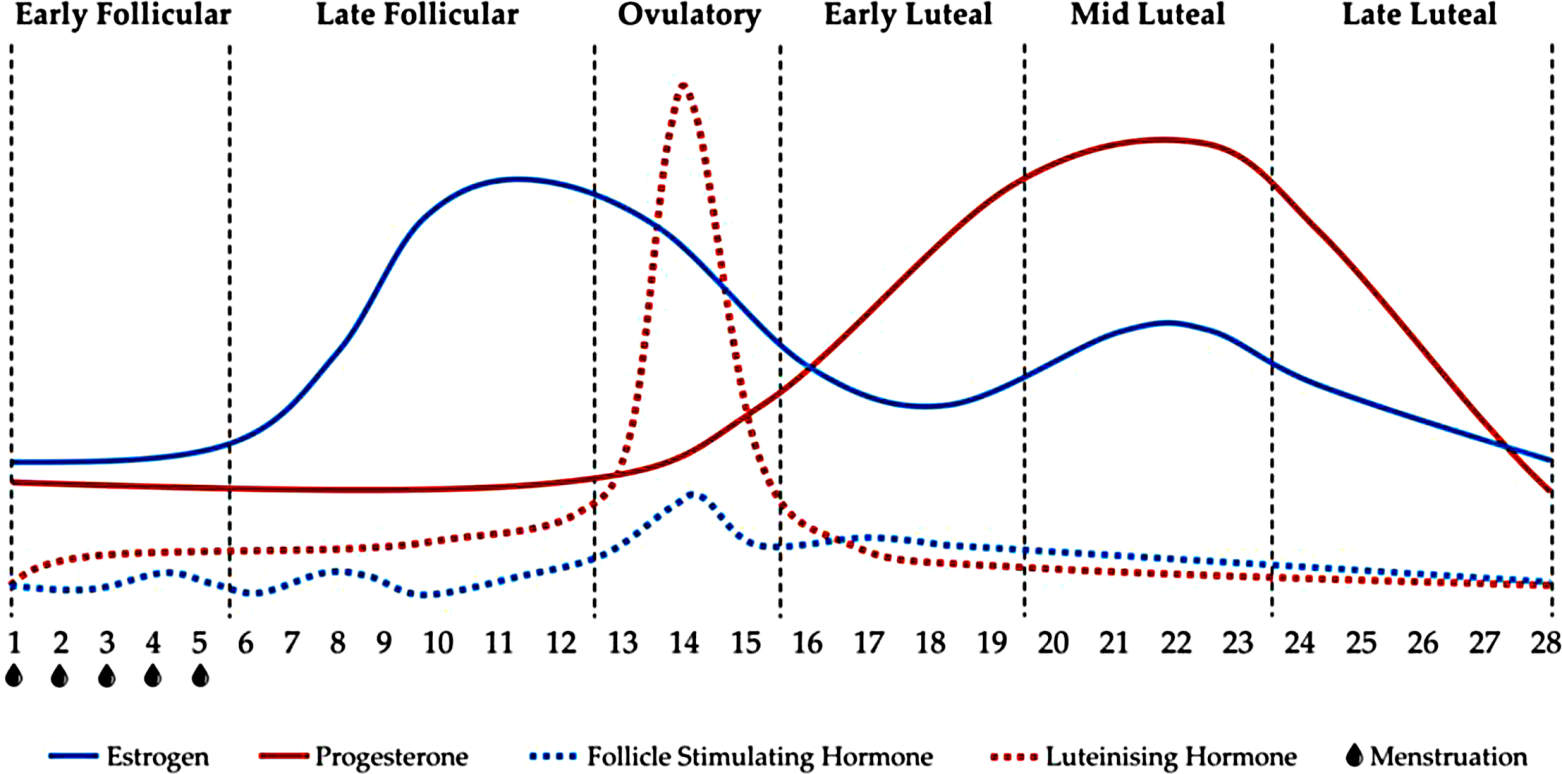
Hormone changes during different phases with a 28-day MC as an example (10).

The MC is a process wherein the endometrium periodically and orderly sheds under the interaction of hormones produced by the hypothalamus, pituitary gland, and ovaries. The shed endometrial tissue and blood are then discharged from the vagina. The beginning of the MC is the first day of menstruation; the MC is generally 28 days long but varies between 25–35 days due to individual differences (9). The day of ovulation is approximately the 14th day. The MC is divided into the follicular phase (FP) and luteal phase (LP) on the basis of ovulation and further divided into subphases (taking a 28-day MC as an example): early follicular (EF) (days 1–5), LF (days 6–12), ovulatory (OVU) (days 13–15), early luteal (EL) (days 16–19), midluteal (ML) (days 20–23), and late luteal (LL) (days 24–28) (10). The MC is regulated by the hypothalamus–pituitary–ovarian axis. The sex hormones related to the MC are mainly estrogen, progesterone, follicle-stimulating hormone (FSH), luteinizing hormone (LH), and gonadotropin-releasing hormone (GnRH) (9). GnRH is secreted by the hypothalamus. It stimulates or inhibits the secretion of pituitary gonadotropin. LH and FSH are pituitary gonadal axis hormones. LH and FSH act synergistically on theca and granulosa cells in the ovary to stimulate the synthesis of gonadal steroids and peptides and formation of ovarian follicles (9). Estrogen and progesterone are the main sex hormones. Hormone fluctuations during different phases are shown in **Figure 1** (10).

**Figure 2:**
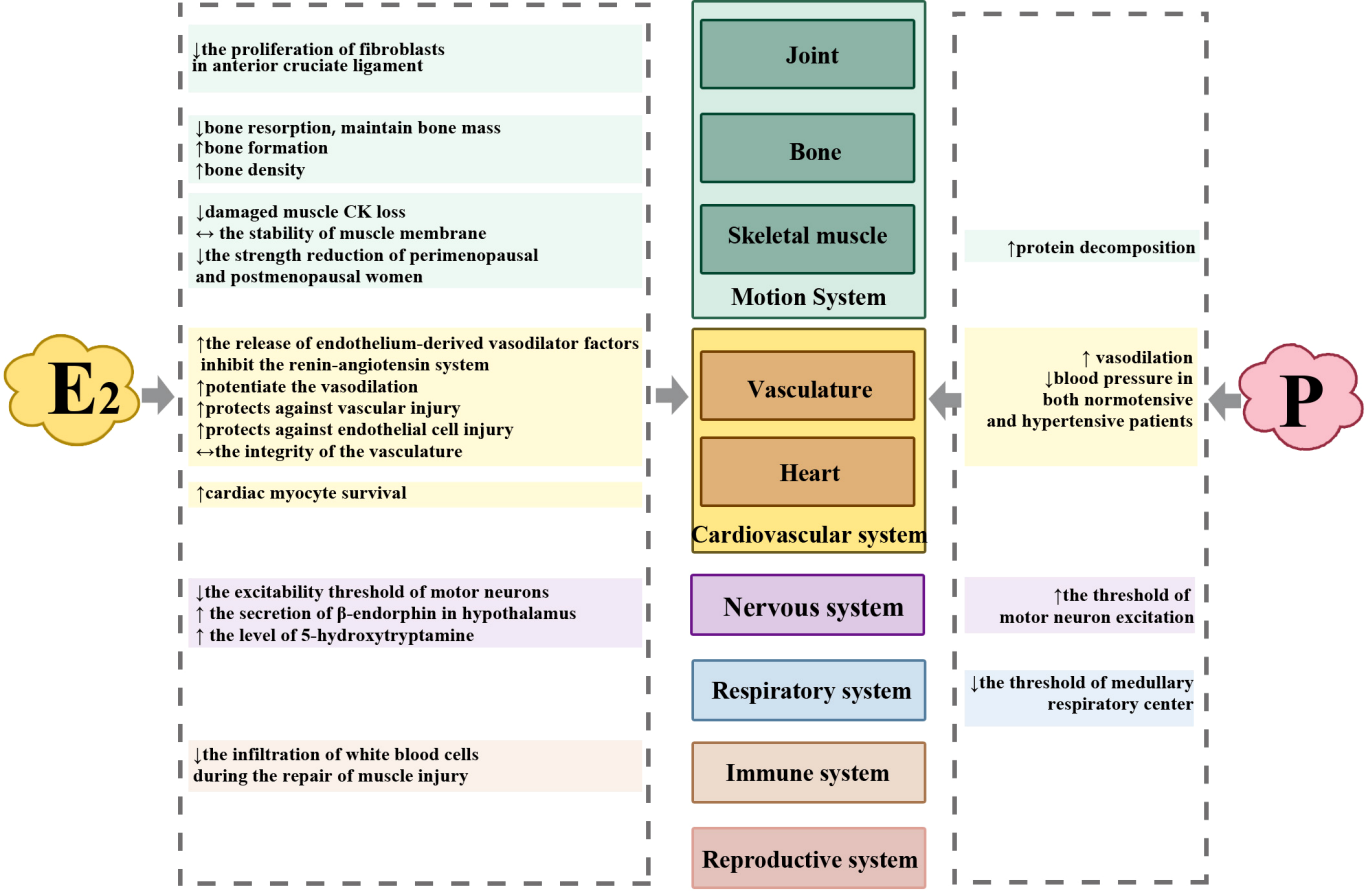
Differences in the effects of estrogen and progesterone on the body system. ↑ = increases; ↓ = inhibition or reduce; ↔ = maintain.

During a MC, follicles (one follicle contains an egg) develop continuously in the FP, and a small amount of estrogen and progesterone are secreted in the ovary during EF. As the follicles gradually develop, the levels of estrogen and progesterone continue to increase. When the secretion of estrogen peaks, the hypothalamus secretes GnRH, and GnRH stimulates the pituitary gland to secrete FSH and LH. After the LH peaks during LF, the mature follicle ruptures, and eggs are released into the uterus to complete ovulation. EL occurs after ovulation, and ruptured follicles turn into the corpus luteum, secreting progesterone and a small amount of estrogen. During ML, estrogen reaches a second low peak, and progesterone also peaks to prepare for the implantation of fertilized eggs. If eggs are not fertilized, the corpus luteum degenerates during the LL, the secretion of estrogen and progesterone gradually decreases, the endometrium sheds, and the next cycle occurs (9). Given that different individuals have different ovulation times, large individual differences in OVU exist, as illustrated in **Figure 2**.

Estrogen is a steroid hormone. It mainly includes estradiol, estrone, and estriol, which are secreted by the ovary. Its receptors are widely distributed in the uterus, vagina, breast, pelvic cavity, skin, bladder, urethra, bone, and brain. Therefore, estrogen can not only promote and maintain female reproductive organs and secondary sexual characteristics, it can also affect the cardiovascular system (including blood pressure, heart rate and rhythm, vascular blood flow, body fluid balance, vascular tension, and cardiovascular response to stress), respiratory system, substrate metabolism, cognition and emotion, and even the brain itself (5–7). It can promote skeletal muscle formation. Hormone replacement therapy with exogenous estrogen can reduce the decrease in muscle strength in perimenopausal and postmenopausal women (4) Progesterone can affect the regulation of body temperature; respiratory system; and even the choice and use of energy, such as the promotion of protein catabolism (5).

The two sex hormones have different functions, and the effects of their interactions on different physiological processes may be different. Both have a potential effect on exercise performance through their influence on individual systems. **Figure 1** shows that the difference between estrogen and progesterone levels is mainly observed during EF (low estrogen and low progesterone), LF (high estrogen and low progesterone), and ML (high estrogen and high progesterone). Therefore, the effects of EF, LF, and ML on exercise performance become increasingly complicated, and more studies have focused on these three phases than on other phases (10).

### 1.2 Accurately framing methods for confirming MC phases

At present, several methods for confirming MC phases (**Table 1**) exist: vaginal ultrasound scans can directly distinguish whether ovulation is occurring to determine whether the MC is in the OVU phase. However, due to its invasiveness, expensive equipment, and high technical requirements, it is not accepted by all subjects and researchers. Determining the MC phase on the basis of dates is simple and easy. This method is conducted on the premise that all subjects experience regular MCs and ovulation. The specific phase is estimated by determining the number of days in the whole cycle. If the MC is normal but the LP is shorter than 9 days, determining whether ovulation has occurred on the basis of dates is impossible, and LP deficiency (LPD) cannot be excluded. In addition, the change rate of the number of days in the FP is higher than that in the LP^[9]^. Phase estimation is also prone to errors. Confirming the MC phase on the basis of the change in basal body temperature (BBT) is also a common method. The morning body temperature of subjects is measured by a thermometer with an accuracy of 0.05°C. In general, body temperature fluctuates at approximately 36.5°C during the FP, reaches its lowest point on ovulation day, rises by 0.3°C to 0.5°C during the LP, and is maintained for 12 to 16 days (9). However, body temperature is easily affected by numerous factors, such as mood, environment, sleep disorders, and temperature. Therefore, the accuracy of this method is inadequate. MC phase can also be determined on the basis of urine luteinizing hormone (LH). This method is usually started on the eighth day of the MC (or the entire MC can be considered as the collection period). The morning urine of the subjects is collected, and the LH peak is determined by using a urine LH test paper, which shows positive results, or a laboratory test. Ovulation usually occurs 14 h to 26 h after the LH peak and lasts for 24–36 h. Some studies have shown that the incidence of LPD is as high as 30% in subjects with positive urine LH test strips (11). In addition, women with luteinized unruptured follicle syndrome may also have a normal MC and LH surges but may not have normal ovulation because the follicle cannot be discharged and follicular luteinization may occur (12). The MC is characterized by fluctuations in estrogen and progesterone. The main ways for determining the MC phase include measuring these two hormones in saliva and blood. The advantage of the saliva test is that it is noninvasive and convenient. However, sex hormones show fluctuations in saliva. Salivary estrogen fluctuates within a 60–90 min cycle (13). Moreover, the level of hormones in saliva accounts for a small amount of total estrogen in serum (14): estrogen and progesterone account for only 1%–2% and 1-3% due to their small amounts. The small differences in hormones at different times have an effect on the accuracy of the results. The serum hormone test method is more accurate than the saliva test method and is considered as the gold standard for determining the MC phase. In this method, venous serum samples (approximately 8 ml) are collected after the same time of fasting in the morning, centrifuged and stored at −80°C, and then detected by using ELISA kits or laboratory tests. However, this method has disadvantages, and continuous blood collection throughout the MC is hindered in actual operation. The use of a single method has certain advantages and disadvantages. For example, vaginal ultrasound scanning or urine LH can only confirm OVU and can only estimate other phases. Therefore, Schaumberg et al. (11) proposed a three-step method for the determination of MC phases. Integrating three methods is recommended. First, the bleeding time and menstrual days of the first 3 months are recorded and counted. After EF, a urine LH test paper strip is used for determination. If it is positive, OVU is occurring. Blood is collected from the 6th day to the 12th day after ovulation with an interval of 1 day or 2 days. This method minimizes intrusion and damage and can accurately confirm OVU and ML. The accuracy of the MC phase determination method affects the accuracy of the analysis of the difference in exercise performance. During preparation for competition, the use of the blood hormone method to determine the MC phase is recommended if the time is sufficient because it is highly accurate. Before the competition, the three-step method, which combines tests based on dates, urine, and blood, is recommended to determine the MC phase quickly with minimal invasiveness and regulate the MC.

**Table 1:** Methods for confirming MC phases.

Methods	Phase	Advantages	Disadvantages
Transvaginal ultrasound scan	OVU	Directly determines whether ovulation is occurring	Expensive equipment, invasive, and not easily accepted by subjects.
Date	EF	Simple, noninvasive.	Cannot rule out luteal insufficiency; errors in inconsistent cycles.
BBT	OVU	Simple, noninvasive.	BBT is susceptible to changes caused by many factors.
Urine LH	OVU	Indirectly distinguish whether ovulation is occurring, noninvasive.	LH surges may occur in anovulatory women.
Salivary estrogen and progesterone	Each phase	Simple, noninvasive.	Sex hormones in saliva fluctuate and are present at low proportions.
Blood estrogen and progesterone	Each phase	Gold standard.	Invasive.

## 2 Methods

A literature search was conducted in the following databases Web of Science, SpringerLink, PubMed Embase, Medline, BMJ (final search 1 November 2025). Keywords included athlete, MC, exercise performance, physical fitness, strength, and menstrual symptoms. The search strategy contained terms related to the MC and performance. A summary table of all included literature information in this study is available in the Supplementary Materials (**Supplementary Table 1**) and an overview of the search and screening process is provided in **Figure A**.

Results from the database searches were imported in EndNote X9 (Clarivate Analytics), where duplicates were removed before screening occurred. One author conducted article screening to identify relevant articles, first based on whether the title and abstract was related to exercise performance over the menstrual cycle, followed by a full-text screening. Articles were included in this narrative review if they were published in English and included populations of women. Participants were considered athletes if they were referred to solely as “athletes” or if specific competitive sports were indicated by the authors. Participants must also have been described as Participants must also have been described as eumenorrheic or having regular menstruation and were not currently using any form of hormonal contraceptives. No exclusion criteria were applied based on participants’ age.

Articles meeting the inclusion criteria must also have reported objective test results of participants, including outcomes from exercise performance tests (maximal tests for measuring exercise performance, such as countermovement jump tests, time trials, or maximal voluntary contraction tests) and applied physiological tests (maximal or submaximal tests for measuring physiological variables related to exercise performance, such as maximal oxygen uptake tests).

Articles must also have compared exercise performance in at least two phases of the MC. To standardize he results from the included articles, where possible, the phases were defined using the MC model established by Redman LM et al. (17) consisting of the early follicular (days 1-5), late follicular (days 6-12), ovulatory (days 13-15), early luteal (days 16-19), mid luteal (days 20-23) and late luteal (days 24-28) phases.

After relevant articles were identified, one author extracted the following information, presented in **Tables 2**, **3**, **4**: Author, year of publication, participant information (sample size and sport level), phases investigated, and the findings of the study. A narrative review was then conducted to summarize the findings and identify the gaps within the literature.

**Table 2:** Related research on aerobic exercise ability at different phases of menstrual cycle.

Research object sports level	Author (Year)	Subject	Phase	Time phase confirmation method	Aerobic performance test method	Conclusion (Differences between phases)
Untrained eumenorrheic women	Gerhard Smekal et al. (2007) (18)	Healthy females n=19	FP and LP	Date, basal body temperature	VO2max	NO
	Teresa M.Deanet al. (2003) (19)	Healthy females n=8	EF, LF and ML	Date, basal body temperature	VO2max and LT	NO
	Janse De Jonge et al. (2012) (16)	Healthy females n=12	EF and ML	Date, basal body temperature (oral), blood progesterone	VO2max	NO
	Tom D.Brutsaert et al. (2002) (20)	Students n=30	LF and ML	Date, saliva progesterone	VO2max	LF better than ML
	Leanne M.Redman et al. (2003) (17)	Sedentary women n=27	FP and LP	Date, urine luteinizing hormone, post-mortem blood progesterone	VO2max and LT	NO
	Dan Gordon et al. (2018) (15)	Recreationally active women n=12	EF, LF, ML and LL	Salivary estradiol, progesterone	VO2max	NO
	Jessica A.Freemas et al. (2021) (21)	Recreationally active women n=12	LF and ML	Basal body temperature, urine luteinizing hormone, salivary progesterone	Ventilation volume per minute, average power	LF better than ML
Athlete	Mariko Nakamura et al. (2021) (24)	Middle distance running, archery, football, skiing, canoeing, skiing, short track and fencing athletes n=10	EF and ML	Date	VO2max and HRmax	NO
	Federica Pisapia et al. (2019) (29)	Athletes n=9	FP and LP	Date	Yo-Yo IET	FP better than LP
	Beatriz Lara et al. (2020) (22)	Triathletes n=13	EF, LF and ML	Date, Basal body temperature (ear)	VO2max	NO
	Ross Julian et al. (2017) (27)	Football players n=9	EF and ML	Date, post-mortem blood estradiol and progesterone	Yo-Yo IET	FP better than ML
	Sille Vaiksaar et al. (2011) (23)	Rowers n=24	LP and ML	Date, post-mortem blood estradiol and progesterone	VO2max	NO
	Jacky J. Forsyth et al. (2005) (26)	Endurance athlete n=7	LP and ML	Date, basal body temperature (oral), urinary luteinizing hormone, post-mortem blood estradiol and progesterone	LT	LF better than ML
	Mohamed TouNSi et al. (2018) (28)	Football players n=11	EF, LF and ML	Date, blood progesterone	Yo-Yo IRT	NO
	Lebrun et al. (1995) (25)	Running, cycling, squash, triathlon, skiers n=16	EF and ML	Basal body temperature, blood progesterone	VO2max	EF better than ML
	Ahmed Graja et al. (2022) (30)	Handball players n=25	LF, ML and LL	Date, urine luteinizing hormone, blood estradiol and progesterone	Intermittent sprinting	LF and ML better than LL

**Table 3:** Related research on anaerobic exercise ability at different phases of menstrual cycle.

Research object sports level	Author (Year)	Subject	Phase	Time phase confirmation method	Aerobic performance test method	Conclusion (Differences between phases)
Untrained eumenorrheic women	Nilsel Okudan et al. (2005) (36)	Sedentary women n=15	LF, OVU and ML	Date	Wingate Test	No
	Shazlin Shaharudin et al. (2011) (37)	Students n=12	LF and ML	Date, basal body temperature (oral), blood progesterone	Maximum Oxygen Deficit Accumulation Test	No
Athlete	Mariko Nakamura et al. (2023) (41)	Middle distance running, archery, football, skiing, canoeing, skiing, short track and fencing athletes n=10	EF and ML	Date	Wingate Test	No
	Aysegul Yapici-Oksuzoglu et al. (2021) (42)	Basketball, futsal, volleyball players n=15	EF and OVU	Date	Wingate Test	The peak power and average power of OVU are better than EF.
	Christine M.Miskec et al. (1997) (40)	Colloge rugby players n=17	EF and LP	Date, basal body temperature (rectal)	Wingate Test	No
	Beatriz Lara et al. (2020) (39)	Triathletes n=13	EF, LF and ML	Date, urine luteinizing hormone, basal body temperature (ear)	Wingate Test	No
	Lebrun et al. (1995) (25)	Running, cycling, squash, triathlon, skiers n=16	EF and ML	Basal body temperature, blood progesterone	Constant-load Test	No

**Table 4:** Related research on the maximum strength at different phases of the menstrual cycle.

Research object sports level	Author (Year)	Subject	Phase	Time phase confirmation method	Aerobic performance test method	Conclusion (Differences between phases)
Untrained eumenorrheic women	Patrick Rodrigues et al. (2019) (54)	Healthy females n=12	EF, LF and LL	Date	Lower body/Maximum voluntary contraction	EF better than LL.
	Tasmektepligil M.Y. et al. (2010) (63)	Sedentary women n=30	EF, LF and LL	Date	Upper body/Maximum voluntary isometric contraction	EF and LF better than LL
	X.A.K.Janse de Jonge et al. (2001) (44)	Healthy females n=19	EF, LF and LP	Basal body temperature	Lower body/Isokinetic muscle strength test	No
	Hiraku Nagahori et al. (2022) (49)	Healthy females n=16	EF and OVU	Date, basal body temperature	Lower body/Isokinetic muscle strength test	No
	Linda Ekenros et al. (2013) (52)	Healthy females n=12	EF, OVU and ML	Date, urine luteinizing hormone	Upper body/Maximum voluntary isometric contraction	No
					Lower body/Isokinetic muscle strength test	ML better than EF
	John P.Abt et al. (2007) (46)	Recreationally active women n=10	EF, OVU and ML	Date, urine luteinizing hormone	Lower body/Isokinetic muscle strength test	No
	K. J. Elliott et al. (2005) (47)	Healthy females n=21	EF and ML	Date, urine luteinizing hormone	Lower body/Maximum voluntary isometric contraction	No
	Elliott K. J. et al. (2003) (51)	Healthy females n=16	EF and ML	Date, basal body temperature (oral), urine luteinizing hormone	Upper body/Maximum voluntary isometric contraction	No
	Cabre H.E et al. (2024) (50)	Recreationally active wome n=10	FP and LP	Date, basal body temperature, urine luteinizing hormone, salivary hormone	Upper body/Maximum voluntary isometric contraction	No
					Lower body/Maximum voluntary isometric contraction, vertical jump height	No
	Paul Ansdell et al. (2019) (55)	Healthy females n=15	EF, OVU and ML	Date, post-mortem blood estradiol and progesterone	Lower body/Maximum voluntary contraction	OVU better than EF and ML
	K. Kubo et al. (2009) (45)	Healthy females n=8	EF, OVU and ML	Date, basal body temperature, postmortem blood estradiol and progesterone	Upper body/Maximum voluntary isometric contraction	No
	Melissa M.Montgomery et al. (2010) (48)	Healthy females n=74	EF and EL	Date, blood estradiol and progesterone	Upper body/Maximum voluntary isometric contraction	No
	Phillips S. K. et al. (1996) (53)	Healthy females n=10	FP and OVU	Date, basal body temperature, urine luteinizing hormone, blood estradiol	Upper body/Maximum voluntary isometric contraction	FP better than OVU
Athlete	Blanca Romero-Moraleda et al. (2019) (56)	Triathletes n=13	EF, LF and ML	Date	Lower body/Half squat 20, 40, 60 and 80%1RM	No
	Otaka M et al. (2018) (58)	Tennis players n=10	EF, LF, OVU and ML	Date	Lower body/Isokinetic muscle strength test	No
	Marília dos SaNToS aNdrade et al. (2017) (60)	Footballers n=26	FP and LP	Date	Lower body/Isokinetic muscle strength test	LP better than FP (The ratio of hamstring strength to quadriceps strength of non-dominant leg)
	Tasmektepligil M.Y.et al. (2010) (63)	Athletes n=30	EF, LF and LL	Date	Upper body/Maximum voluntary isometric contraction	EF and LF better than LL
	Sima Forouzandeh Shahraki et al. (2020) (62)	College athletes n=15	EF, OVU and ML	Date, urine luteinizing hormone	Upper body/Maximum voluntary isometric contraction	OVU better than EF and ML
	Jay Hertel et al. (2006) (57)	College athletes n=14	EF and OVU	Date, urine luteinizing hormone	Lower body/Isokinetic muscle strength test	No
	Quigley T et al. (2025) (59)	Soccer players n=8	EF and OVU	Date, urine luteinizing hormone	Lower body/Isokinetic muscle strength test	NO
	Lebrun et al. (1995) (25)	Running, cycling, squash, triathlon, skiers n=16	EF and ML	Basal body temperature, blood progesterone	Lower body/Isokinetic muscle strength test	No
	Ahmed Graja et al. (2022) (30)	Handball players n=25	LF, ML and LL	Date, urine luteinizing hormone, blood estradiol and progesterone	Lower body/Maximum voluntary contraction	LF and ML better than LL
	Phillips S. K. et al. (1996) (53)	Athletes n=12	FP and OVU	Date, basal body temperature, urine luteinizing hormone, blood estradiol	Upper body/Maximum voluntary isometric contraction	FP better than OVU

## 3 Differences in exercise performance at different MC phases and possible mechanisms

### 3.1 Aerobic performance

Sixteen relevant studies were included, a summary of the findings and characteristics of the included studies is provided in **Table 2**. In a study on the female population, Gordon et al. (15) found that in women with normal MC, hormone fluctuations did not affect oxygen uptake (VO2max), maximum heart rate, and maximal stroke volume; this finding was consistent with previous results (16–19). A study on LT (17–19) also found no effect. However, different views also exist. Some studies determined the MC phase by using salivary progesterone and found that aerobic performance during ML was lower than that during other phases. For example, Brutsaert et al. (20) found that VO2max during ML had reduced by 2% relative to that during LF. Freemas et al. (21) discovered no change in absolute VO2 and volume ventilation and HR by in 8 km bicycle test. However, compared with that during LF, average power was worse during ML, and negative emotions and RPE before exercise increased.

A study on female athletes showed that the VO2max of triathlon (22), rowing (23), middle-distance running, and football athletes (24) was unaffected by the MC. However, early studies discovered fluctuations in VO2max. For example, in 1995, Lebrun et al. (25) demonstrated that absolute VO2max was greater during EF than during ML but found no change in relative VO2max and time to exhaustion. By conducting the lactate threshold (LT) test on seven endurance athletes in 2005, Forsyth et al. (26) discovered that the LT level during LF was higher than that during ML.

In addition to maximum VO2max and LT, aerobic performance can be estimated by intermittent endurance tests (IETs). The YOYO test showed that high-level football players performed on IET better during EF than during ML (27) and their intermittent recovery test (IRT) results did not change (28). The YOYO test of nine ordinary athletes also found that IET performance during the FP was better than that during the LP (29). Twenty 5 s power bicycle sprint tests were performed on handball players. The percentage of peak power reduction from the first to the 20th cycle was greater during LL than during LF and ML (30).

The inconsistency in findings from studies on women’s aerobic performance in relation to body mass and exercise may be associated with exercise continuity. The results of continuous exercise on maximal oxygen uptake only partially supported that aerobic performance was affected by the MC, whereas tests on intermittent exercise showed that intermittent endurance and sprint performance were affected by the MC. In general, studies indicating that aerobic performance is affected by MC tend to support that aerobic performance is higher during the FP than the LP. Although progesterone can increase excitability by reducing the threshold of the medullary respiratory center (31), the increase in ventilation affects respiration. In substrate metabolism, estrogen and progesterone have different effects on sugar storage and utilization and lipid metabolism. In terms of glycogen storage, estrogen promotes insulin sensitivity and may increase glycogen storage, whereas progesterone promotes insulin resistance (32). In glycogen utilization, estrogen reduces carbohydrate oxidation rates by reducing muscle glycogen utilization and muscle glucose uptake (33). In lipid metabolism, estrogen can increase the availability of free fatty acids as energy substrates during exercise and promote lipid oxidation in skeletal muscle, whereas progesterone antagonizes the effect of estrogen by inhibiting lipid oxidation (34). Therefore, high levels of estrogen during the FP may improve endurance performance by changing carbohydrate, fat, and protein metabolism. Although progesterone is antagonistic, its level during the FP is low. In addition to estrogen and progesterone, exercise intensity, nutritional status, exercise level, and individual hormone differences all affect the rate of substrate oxidation. This influence may explain the differences in the results of aerobic performance studies. In addition, studies have demonstrated that glucose intake can minimize the effect of MC on glucose metabolism (35) and reduce the effect on aerobic performance.

### 3.2 Anaerobic performance

Sixteen of the included studies examined at least one anaerobic performance outcome; most of these studies (n = 6) determined that MC phase had no effect and only one studies demonstrated at least one anaerobic performance outcome fluctuated with MC phase (**Table 3**).

Studies on the female population showed that Wingate peak power, average power (36), and maximal accumulated oxygen deficit (37) did not significantly differ between the FP and ML. However, after repeated sprints during the LP, serum progesterone significantly reduced. Nonathletes subjected to high-intensity tests during the LP exerted poor emotional and perceptual efforts (38), resulting in a low level of motivation and ultimately different research results.

Studies on female athletes (triathlon (39), rowing (40), middle- and long-distance running, football, and skiing (41)) found no significant difference in Wingate anaerobic power test results between the EF and ML phases. In addition, the results of lactic acid accumulation, average power (40), and constant load test (25) after five groups of 15-second bicycle sprints (interval 2 minutes) were unaffected by the MC. However, Yapic et al. (42) tested 15 athletes and found that Wingate peak power and average power results were better during OVU than during EF.

Anaerobic exercise derives energy through phosphate and anaerobic glycolysis in muscle. There is a lack of research examining the impact of the menstrual cycle (MC) on phosphate utilization. However, Harber et al. (43) compared endurance athletes with amenorrhea and normal MC and found that after plantar flexion training, the recovery of creatine phosphate in athletes with amenorrhea was slow, indicating that the hormone level of the ovary may affect the recovery of phosphate.

The inconsistent results of anaerobic performance research may be related to the determination method of exercise levels and MC phases but tend to support the conclusion that anaerobic performance is unaffected by the MC. Untrained eumenorrheic women has not received long-term anaerobic training, and their level of anaerobic performance itself is low. Therefore, finding differences in studies conducted during different MC phases is difficult. Among four-item studies on athletes (25, 39–41), only the work of Lebrun et al. (25) used blood hormones to determine the MC phase. Given that the methods based on dates, body temperature, and urine utilized in other studies are inaccurate, different conclusions were obtained. The use of blood hormones is recommended in future experiments to increase the accuracy of conclusions.

### 3.3 Muscle strength

#### 3.3.1 Maximal strength

A total of twenty-one relevant studies were incorporated, with a synopsis of their key findings and attributes presented in **Table 4**. In studies on untrained eumenorrheic women, six scholars tested the flexion and extension of the knee joint of the lower body through isokinetic muscle strength (44, 46, 49) or maximum voluntary contraction (45, 47, 48, 50) tests and found that the maximum strength of the lower body was unaffected by the MC. No significant difference in upper body grip strength at different times was found (51, 52). However, two scholars believe that maximum strength fluctuates during the menstrual period (53, 54). Phillips et al. (53) believed that the upper body grip strength is higher during the FP than during other phases. Rodrigues et al. (54) found through the 45° pedal test of the lower body that the maximum strength of the lower body during EF was higher than that during LL, and the maximum strength of the lower body was greater during the premenstrual period than during the postmenstrual period. The results of both studies suggested that strength performance is best during the FP. However, different conclusions were also reached (52, 55). Ansdell et al. (55) believed that the muscle strength of the lower body was highest during OVU. In the isokinetic test on the knee joints of ordinary college students, Ekenros et al. (52) found that strength during EF was less than that during ML.

The results of research on female athletes are also inconsistent. Some studies have shown no significant change in the maximum strength of the lower body in different time phases (25, 56–60). Some studies have also illustrated that the maximum voluntary contraction strength of the knee extensor muscle group is lower during LF and ML than during LL^[30]^. Dos Santos et al. (60) tested the knee muscle strength of football players during the FP and LP and found that the ratio of hamstring to quadriceps strength of nondominant legs was greater during the LP than during the FP but found no significant difference in dominant legs. In terms of upper body muscle strength, Shahraki et al. (61) tested upper body shoulder strength (abduction, internal rotation, and external rotation) and reported that strength was higher during OVU than during EF and ML and grip strength was best during the FP (62).

The results of studies on the effect of MC on maximum strength showed that when estrogen level was high, maximum strength also increased. Maximum strength was worst during the LP likely because in the physiological cycle, estrogen gradually decreased after OVU, whereas progesterone gradually increased and peaked during ML. The specific mechanism of the effect of the fluctuating relationship between estrogen and progesterone on maximum strength remains unclear. However, the two hormones can affect the physiological factors of maximum strength, such as nerve excitability and the contraction coupling of muscle fibers. Estradiol and progesterone have opposite effects on nerve excitation. Specifically, estradiol can increase glutamate-mediated neuronal excitability and reduce the neuronal excitation threshold; the neurosteroid metabolites of progesterone can bind to the γ-aminobutyric acid (GABA) receptor complex, enhance its activity via an allosteric mechanism, reduce neuronal excitability, and elevate the neural excitation threshold (63). In terms of muscle contraction, estrogen promotes the improvement in myosin function by acting on estrogen receptors, thereby affecting the binding of myosin to actin (64). Experiments on rodents and humans have shown that estrogen can promote strength by changing muscle mass (64). In addition, female endogenous testosterone (T) also fluctuates during the MC. Such fluctuations are manifested by the gradual increase in serum total and free T during the FP; serum total T and free T then peak during OVU and gradually decrease during the LP (65). A study examined the relationship between endogenous testosterone (T) concentration in females and muscle mass, strength, and surface muscle performance (66). It found that quadriceps muscle mass and maximal stretching strength increased as levels of free testosterone or bioavailable testosterone rose (66). During the FP, estrogen and T are high, and the positive effects of both hormones on nerves and muscles account for the optimal maximum muscle strength during this phase.

Exploring the potential of physiological cycle characteristics to inform strength training planning may hold practical value. According to Kissow et al. (67), resistance training during the FP was observed to promote muscle strength and increase muscle volume to a greater extent than during the LP in their study. In training practice, adjusting the frequency of strength training during the FP might be a viable approach to explore for optimizing training outcomes.

#### 3.3.2 Power strength

Studies on untrained eumenorrheic women found no significant differences in various jump performance metrics across menstrual phases: squat jump (SJ) and multiple jump tests (maximum power and height) during EF, LF, and ML (68); countermovement jump (CMJ), drop jump (reaction strength index) (69), and vertical jumps (height and power) during EF, LF, and LL (70); and single-leg jump distances during EF and LP (52). However, different results were also obtained (42, 69). Aysegul et al. (42) showed that CMJ performance during OVU was better than that during EF, and Felipe et al. (69) reported that SJ height during the LP was higher than that during EF.

The results of studies on female athletes are also inconsistent (62, 71, 72). Tsolakis et al. (71) used Boso Ergo jump test equipment to test the CMJ, SJ, and repeated jump performances of 10 international fencing athletes and found no significant differences in lower body power strength during EF, LF, and LL; the CMJ and SJ test results of handball players during EF, LF, and the LP also showed no significant differences (72). However, when Tasmektepligil et al. (62) used Takei jump test equipment on 30 athletes (including basketball, football, and judo) during the same three phases, they found that CMJ was better during LL than during EF.

Research exploring the relationship between lower-body power and the MC remains limited. Power strength is not only affected by physiological factors, such as age, weight, body fat, muscle volume, and nervous system regulation, but also by training factors. Various factors must be included and comprehensively considered when considering the influence of MC on power strength.

## 4 Related factors affecting the difference in exercise ability during different MC phases

### 4.1 MC-related symptoms

The MC is a unique biological rhythm of women. The psychological and physiological symptoms associated with menstruation have a negative effect on the daily life and work of women. More than 100 potential psychological changes, such as anxiety and depression, exist. Physiologically, MC manifests as lower abdominal colic, abdominal distension, fatigue, drowsiness, and severe premenstrual syndrome (PMS) (9). Martin et al. (73) found that 77% of female athletes (N = 430) had conditions, such as pain, cramps, headache, or migraine and even PMS. Heavy menstrual bleeding (HMB) can also lead to fatigue, anxiety, and other negative emotions and has a negative effect on life. A survey found that the incidence of HMB in the sports population is higher than that in the general population, and the incidence of HMB in elite athletes reached 37% (74). The incidence of exercise-associated menstrual disorders in female athletes has also increased annually (75).

In a cross-sectional survey of elite female athletes by Ergin et al. (76), 70% of female athletes (N = 430) reported that EF-related symptoms affected their participation in competition or training. A four-phase follow-up survey of six college athletes (77) revealed that self-perception strength and speed were poor during ML and LL, and explosive power was poor during EF, ML, and LL. The above results in elite or ordinary athletes support that women perform worst during EF and LL and better during other phases. This finding is consistent with the proneness to fatigue, drowsiness, and pain during EF and LL.

### 4.2 Body weight and composition

Many women state that their weight fluctuates throughout the MC. This condition may affect exercise performance. Although some studies on female athletes and untrained eumenorrheic women showed that no remarkable change in body weight and body fat occur throughout the MC (25), some have shown that body weight decreases gradually from EF to LF and then increases continuously from EF to the LP. This change may be due to the increase in extracellular water (78). It may also be due to the increase in progesterone and decrease in insulin during the LP, resulting in increased appetite and food intake (79). Hormonal fluctuations during the MC have been suggested to not affect weight and body water changes; it may instead affect distribution rather than the body’s retention or exclusion of water (80).

### 4.3 BBT

Most studies support that the BBT fluctuates during the MC, that is, the BBT is high during the LP likely in relation to the increase in progesterone. Studies have shown that estrogen and progesterone may interact in a complex manner to regulate body temperature through the central and peripheral systems. Progesterone acts on thermosensitive neurons in the preoptic area of the hypothalamus to increase heat production or reduce heat loss, whereas estrogen promotes heat loss or heat production reduction. Estrogen and progesterone may also feed back to the hypothalamus through the effect of the peripheral vasomotor state to further regulate body temperature (81). However, the BBT during the LP is only 0.3°C–0.5°C higher than that during other phases. Studies have illustrated that sufficient warm-up activities can offset the difference in BBT caused by different phases without affecting exercise performance during different phases (82).

## 5 Attitudes of athletes and coaches toward MC

Although reports related to the female MC are increasing and deepening, the MC may remain an obscure term for the eumenorrheic women. The MC is considered a shameful and embarrassing topic. A survey shows that stigma continues to surround the MC. One in four women states they are overwhelmed at the time of menarche, and 48% of women say they are ashamed when they talk about the MC (83). This situation also exists in female athletes. According to a survey of elite athletes, some athletes are reluctant to communicate MC problems with their coaches or medical staff because they are ashamed or because of gender differences and believe that communicating with them will not help (84).

Communication between coaches and female athletes is equally important. Most coaches emphasize sports-related techniques and tactics, teaching methods, athlete management, and nutrition and pay little attention to the MC of athletes and its effect on exercise performance (85). The attitudes of male and female coaches toward the MC differ. Male coaches think that the regularity of the MC is unimportant, do not understand relevant knowledge, and find communicating about the MC with female athletes inconvenient (86). Although female coaches have a natural gender advantage in terms of the MC of athletes, most female athletes’ coaches are male (87). This situation increases the difficulty of communication between coaches and female athletes. Coaches’ neglect of the MC-related needs of athletes may lead to the athletes’ inability to leverage their exercise performance fully due to the effect of the MC, which in turn affects competition results. The important role of the coach’s identity in the athlete’s entire sports career is not fully reflected. Therefore, further improving athletes’ own understanding of the MC, improving coaches’ attitude towards athletes’ MC, and increasing communication between coaches and athletes are recommended to reduce the negative effect of the MC on exercise performance.

## 6 Conclusion

Although a considerable proportion of female athletes believe that their exercise performance is affected by the MC, some differences may be due to small sample sizes, lack of blood hormone–based methods to determine the MC phase, poor control of confounding factors, and nonrandomization of experiments. No significant difference in exercise performance during different phases of the MC have been observed in the present study. Existing research examining differences in exercise performance has produced conflicting conclusions. Anaerobic performance generally appears unaffected by variations in MC phases, though ovarian hormones may influence post-exercise energy recovery. Aerobic performance tends to be greater during FP than LP, with glucose intake potentially mitigating the impact of MC-related hormonal fluctuations on this performance. Maximum strength performance tends to be relatively lower during the LP. Nevertheless, the patterns of hormonal fluctuations throughout the MC may offer potential insights for refining strength training approaches to support muscle strength enhancement. The difference in explosive power during different phases of the MC remains controversial.

In recent years, due to the increase in women’s participation in sports, the research on the exercise performance of women has also increased. Many methods for determining the phase of the female MC, an important step in this kind of research, exist. Some inaccuracies, except for blood estrogen and progesterone, were observed. Although some scholars used a combination of methods, adding blood estrogen and progesterone (including *post hoc*) measurement is recommended to enhance accuracy. In addition, current research usually provided a cross-sectional test or questionnaire to untrained eumenorrheic women or athletes. The comparison of the differences between untrained eumenorrheic women and elite athletes is scarce, and whether differences among female athletes at different sports levels exist are unclear. Studies on exercise ability focused on aerobic, anaerobic, and strength parameters, whereas research on speed, sensitivity, and flexibility is lacking. Further research is needed to quantify the exercise performance during different phases of the MC and determine the relevant factors affecting the change in exercise performance to understand women’s exercise performance fully. Such research provides help to athletes in regulating their physiological cycle before games and ensuring their full performance during competition.

Clarifying the effect of MC on various exercise performance is crucial for sports researchers and coaches to formulate training plans rationally, ensure athletes’ physical health, improve exercise performance, and extend sports careers. Therefore, further studying the influence of MC on sports ability and its potential mechanism is necessary. In some cases, the related problems of athletes’ MC should be discussed in the same important position as other training loads and skills that affect exercise performance. The communication between athletes and coaches should be increased, open discussion and analysis should be conducted with a positive attitude, and more women should be encouraged to participate in sports.

## Supplementary material

Supplementary Figure Summary of the search and screening methods implemented in this narrative review.

## References

[1] International Olympic Committee . IOC gender equality review project-women in the olympic movement (2024). Available online at: https://stillmed.olympics.com/media/Documents/Olympic-Movement/Factsheets/Women-in-the-Olympic-Movement.pdf (Accessed March 18, 2024).

[2] Dong H Wang Y Li W Dindin J . Socioeconomic disparities and inequality of mass sports participation: Analysis from Chinese General Social Survey 2010-2018. Front Public Health. (2023) 11:1072944. doi: 10.3389/fpubh.2023.1072944, PMID: 36844848 PMC9948005

[3] Elliott-Sale KJ Minahan CL De Jonge XAKJ Ackerman KE Sipilä S Constantini NW . Methodological considerations for studies in sport and exercise science with women as participants: A working guide for standards of practice for research on women. Sports Med (Auckland N.z.). (2021) 51:843–61. doi: 10.1007/s40279-021-01435-8, PMID: 33725341 PMC8053180

[4] Ssipilä S Finni T Kovanen V . Estrogen influences on neuromuscular function in postmenopausal women. Calcif Tissue Int. (2015) 96:222–33. doi: 10.1007/s00223-014-9924-x, PMID: 25359124

[5] Constantini NW Dubnov G Lebrun CM . The menstrual cycle and sport performance. Clin Sports Med. (2005) 24:e51–82. doi: 10.1016/j.csm.2005.01.003, PMID: 15892917

[6] Boss L Kang DH Marcus M Bergstrom M . Endogenous sex hormones and cognitive function in older adults: A systematic review. West J Nurs Res. (2014) 36:388–426. doi: 10.1177/0193945913500566, PMID: 23996907

[7] Oosthuyse T Strauss JA Hackney AC . Understanding the female athlete: molecular mechanisms underpinning menstrual phase differences in exercise metabolism. Eur J Appl Physiol. (2023) 123:423–50. doi: 10.1007/s00421-022-05090-3, PMID: 36402915

[8] Meignié A Toussaint JF Antero J . Dealing with menstrual cycle in sport: stop finding excuses to exclude women from research. Eur J Appl Physiol. (2022) 122:2489–90. doi: 10.1007/s00421-022-05014-1, PMID: 35984494

[9] Hall JE . Neuroendocrine Control of the Menstrual Cycle, in Yen & Jaffe’s Reproductive Endocrinology (Seventh Edition). Philadelphia: W.B. Saunders (2014) 149–166.e5. doi: 10.1016/C2015-0-05642-8

[10] Carmichael MA Thomson RL Moran LJ Wycherley TP . The impact of menstrual cycle phase on athletes’ Performance: A narrative review. Int J Environ Res Public Health. (2021) 18:1667. doi: 10.3390/ijerph18041667, PMID: 33572406 PMC7916245

[11] Schaumberg MA Jenkins DG Janse de Jonge XA Emmerton LM Skinner TL . Three-step method for menstrual and oral contraceptive cycle verification. J Sci Med Sport. (2017) 20:965–9. doi: 10.1016/j.jsams.2016.08.013, PMID: 28684053

[12] Su H Yi Y Wei T Chang TC Cheng CM . Detection of ovulation, a review of currently available methods. Bioeng Transl. (2017) 2:238–46. doi: 10.1002/btm2.10058, PMID: 29313033 PMC5689497

[13] Chatterton RT Mateo ET Hou N Rademaker AW Acharya S Jordan VC . Characteristics of salivary profiles of oestradiol and progesterone in premenopausal women. J Endocrinol. (2005) 186:77–84. doi: 10.1677/joe.1.06025, PMID: 16002538

[14] Huang T Howse FM Stachenfeld NS Usselman CW . Correlations between salivary- and blood-derived gonadal hormone assessments and implications for inclusion of female participants in research studies. Am J Physiol Heart Circ Physiol. (2023) 324:H33–46. doi: 10.1152/ajpheart.00399.2022, PMID: 36426884

[15] Gordon D Scruton A Barnes R Baker J Pradoet L Merzbach V . The effects of menstrual cycle phase on the incidence of plateau at V˙O2max and associated cardiorespiratory dynamics. Clin Physiol Funct Imaging. (2018) 38:689–98. doi: 10.1111/cpf.12469, PMID: 28906053

[16] Janse de Jonge XA Thompson MW Chuter VH Silk LN Thom JM . Exercise performance over the menstrual cycle in temperate and hot, humid conditionsal. Med Sci sports Exercise. (2012) 44:2190–8. doi: 10.1249/MSS.0b013e3182656f13, PMID: 22776870

[17] Redman LM Scroop GC Norman RJ . Impact of menstrual cycle phase on the exercise status of young, sedentary women. Eur J Appl Physiol. (2003) 90:505–13. doi: 10.1007/s00421-003-0889-0, PMID: 12898264

[18] Smekal G Von Duvillard SP Frigo P Tegelhofer T Pokan R Hofmann P . Menstrual cycle: no effect on exercise cardiorespiratory variables or blood lactate concentration. Med Sci Sports Exerc. (2007) 39:1098–106. doi: 10.1249/mss.0b013e31805371e7, PMID: 17596777

[19] Dean TM Perreault L Mazzeo RS Horton TJ . No effect of menstrual cycle phase on lactate threshold. J Appl Physiol. (2003) 95:2537–43. doi: 10.1152/japplphysiol.00672.2003, PMID: 14600163

[20] Brutsaert TD Spielvogel H Caceres E Araoz M Chatterton RT Vitzthum VJ . Effect of menstrual cycle phase on exercise performance of high-altitude native women at 3600m. J Exp Biol. (2002) 205:233–9. doi: 10.1242/jeb.205.2.233, PMID: 11821489

[21] Freemas JA Baranauskas MN Constantini K Constantin N Greenshields JT Mickleborough T . Exercise performance is impaired during the midluteal phase of the menstrual cycle. Med Sci Sports Exerc. (2021) 53:442–52. doi: 10.1249/MSS.0000000000002464, PMID: 32694375

[22] Lara B Gutierrez-Hellin J Garcia-Bataller A Rodríguez−Fernández P Romero−Moraleda B Coso JD . Ergogenic effects of caffeine on peak aerobic cycling power during the menstrual cycle. Eur J Nutr. (2020) 59:2525–34. doi: 10.1007/s00394-019-02100-7, PMID: 31691019

[23] Vaiksaar S Jürimäe EJ Mäestu J Purge P Kalytka S Shakhlina L . No effect of menstrual cycle phase and oral contraceptive use on endurance performance in rowers. J Strength Cond Res. (2011) 25:1571–8. doi: 10.1519/JSC.0b013e3181df7fd2, PMID: 21399539

[24] Nakamura M Nose-ogura S . Effect of administration of monophasic oral contraceptive on the body composition and aerobic and anaerobic capacities of female athletes. J Obstet Gynaecol Res. (2021) 47:792–9. doi: 10.1111/jog.14613, PMID: 33336549

[25] Lebrun CM Mckenzie DC Prior J Prior JC Taunton JE . Effects of menstrual-cycle phase on athletic performance. Med Sci Sports Exerc. (1995) 27:437–44. doi: 10.1249/00005768-199503000-00022 7752873

[26] Forsyth JJ Reilly T . The combined effect of time of day and menstrual cycle on lactate threshold. Med Sci Sports Exerc. (2005) 37:2046–53. doi: 10.1249/01.mss.0000179094.47765.d0, PMID: 16331128

[27] Julian R Hecksteden A Fullagar HH Meyer T . The effects of menstrual cycle phase on physical performance in female soccer players. PloS One. (2017) 12:e0173951. doi: 10.1371/journal.pone.0173951, PMID: 28288203 PMC5348024

[28] Tounsi M Jaafar H Aloui A aloui A SouiSSi N . Soccer-related performance in eumenorrheic Tunisian high-level soccer players: effects of menstrual cycle phase and moment of day. J Sports Med Phys Fitness. (2018) 58:497–502. doi: 10.23736/S0022-4707.17.06958-4, PMID: 28222573

[29] Pisapia F . Correlation between menstrual cycle and performance. J Phys Educ Sport. (2019) 2019:1972–5. doi: 10.7752/jpes.2019.s5293

[30] Graja A Kacem M Hammouda O Borji R Bouzid MA Souissi N . Physical, biochemical, and neuromuscular responses to repeated sprint exercise in eumenorrheic female handball players: effect of menstrual cycle phases. J Strength Cond Res. (2022) 36:2268–76. doi: 10.1519/JSC.0000000000003556, PMID: 32168179

[31] Williams TJ Krahenbuhl GS . Menstrual cycle phase and running economy. Med Sci Sports Exerc. (1997) 29:1609–18. doi: 10.1097/00005768-199712000-00010, PMID: 9432094

[32] Oosthuyse T Bosch AN . The effect of the menstrual cycle on exercise metabolism: implications for exercise performance in eumenorrhoeic women. Sports Med. (2010) 40:207–27. doi: 10.2165/11317090-000000000-00000, PMID: 20199120

[33] D’Eon TM Sharoff C Chipkin SR Grow D Ruby BC Braun B . Regulation of exercise carbohydrate metabolism by estrogen and progesterone in women. Am J Physiol Endocrinol Metab. (2002) 283:E1046–55. doi: 10.1152/ajpendo.00271.2002, PMID: 12376334

[34] Oosthuyse T Bosch AN . The effect of the menstrual cycle on exercise metabolism implications for exercise performance in eumenorrhoeic women. Sports Med. (2010) 40:207–27. doi: 10.2165/11317090-000000000-00000, PMID: 20199120

[35] Campbell SE Angus DJ Febbraio MA . Glucose kinetics and exercise performance during phases of the menstrual cycle: effect of glucose ingestion. Am J Physiol Endocrinol Metab. (2001) 281:E817–25. doi: 10.1152/ajpendo.2001.281.4.E817, PMID: 11551860

[36] Okudan N Gökbel H Uçok K Baltaci AK . Serum leptin concentration and anaerobic performance do not change during the menstrual cycle of young females. Neuro Endocrinol Lett. (2005) 26:297–300., PMID: 16136023

[37] Shaharudin S Ghosh AK Ismail AA . Anaerobic capacity of physically active eumenorrheic females at mid-luteal and mid-follicular phases of ovarian cycle. J Sports Med Phys Fitness. (2011) 51:576–82., PMID: 22212259

[38] Prado RCR Silveira R Kilpatrick M Pires FO Asano RY . The effect of menstrual cycle and exercise intensity on psychological and physiological responses in healthy eumenorrheic women. Physiol Behav. (2021) 232:113290. doi: 10.1016/j.physbeh.2020.113290, PMID: 33333131

[39] Lara B Gutierrez Hellin J Ruiz-Moreno C Romero-Moraleda B Coso JD . Acute caffeine intake increases performance in the 15-s Wingate test during the menstrual cycle. Br J Clin Pharmacol. (2020) 86:745–52. doi: 10.1111/bcp.14175, PMID: 31747465 PMC7098873

[40] Miskec CM Potteiger JA Nan KL Zebas CJ . Do varying environmental and menstrual cycle conditions affect anaerobic power output in female athletes? J Strength Cond Res. (1997) 11:219–23.

[41] Matsuda T Takahashi H Nakamura M Ogata H Kanno M Shikawa A . Influence of the menstrual cycle on muscle glycogen repletion after exhaustive exercise in eumenorrheic women. J Strength Cond Res. (2023) 37:e273–9. doi: 10.1519/JSC.0000000000004306, PMID: 35836304

[42] Yapici-Oksuzoglu A Egesoy H . The effect of menstrual cycle on anaerobic power and jumping performance. Pedagog Phys Cult Sp. (2021) 25:367–72. doi: 10.15561/26649837.2021.0605

[43] Harber VJ Petersen SR Chilibeck PD . Thyroid hormone concentrations and muscle metabolism in amenorrheic and eumenorrheic athletes. Can J Appl Physiol. (1998) 23:293–306. doi: 10.1139/h98-017, PMID: 9615871

[44] Janse de Jonge XAK Boot CRL Thom JM Ruell PA Thompson MW . The influence of menstrual cycle phase on skeletal muscle contractile characteristics in humans. J Physiol. (2001) 530:161–6. doi: 10.1111/j.1469-7793.2001.0161m.x, PMID: 11136868 PMC2278395

[45] Kubo K Miyamoto M Tanaka S Maki A Tsunoda N Kanehisa H . Muscle and tendon properties during menstrual cycle. Int J Sports Med. (2009) 30:139–43. doi: 10.1055/s-0028-1104573, PMID: 19067277

[46] Abt JP Sell TC Laudner KG McCrory JL Loucks TL Berga SL . Neuromuscular and biomechanical characteristics do not vary across the menstrual cycle. Knee Surg Sports Traumatol Arthros. (2007) 15:901–7. doi: 10.1007/s00167-007-0302-3, PMID: 17364205

[47] Elliott KJE Cable NT Reilly T . Does oral contraceptive use affect maximum force production in women? Br J Sports Med. (2005) 39:15–9. doi: 10.1136/bjsm.2003.009886, PMID: 15618333 PMC1725011

[48] Montgomery MM Shultz SJ . Isometric knee-extension and knee-flexion torque production during early follicular and postovulatory phases in recreationally active women. J Athl Train. (2010) 45:586–93. doi: 10.4085/1062-6050-45.6.586, PMID: 21062182 PMC2978010

[49] Nagahori H Shida N . Relationship between muscle flexibility and characteristics of muscle contraction in healthy women during different menstrual phases. Phys Ther Res. (2022) 25:68–74. doi: 10.1298/ptr.E10173, PMID: 36118784 PMC9437931

[50] Cabre HE Joniak KE Ladan AN Moore SR Blue MNM Pietrosimone BG . Effects of hormonal contraception and the menstrual cycle on maximal strength and power performance. Med Sci Sports Exercise. (2024) 56:2385. doi: 10.1249/MSS.0000000000003524, PMID: 39160762 PMC12666725

[51] Elliott KJ Cable NT Reilly T Diver MJ . Effect of menstrual cycle phase on the concentration of bioavailable 17-beta oestradiol and testosterone and muscle strength. Clin Sci. (2003) 105:663–9. doi: 10.1042/CS20020360, PMID: 12848619

[52] Ekenros L Hirschberg AL Heijne A Fridén C . Oral contraceptives do not affect muscle strength and hop performance in active women. Clin J Sport Med. (2013) 23:202–7. doi: 10.1097/JSM.0b013e3182625a51, PMID: 22948447

[53] Phillips SK Sanderson AG Birch K Bruce SA Woledge RC . Changes in maximal voluntary force of human adductor pollicis muscle during the menstrual cycle. J Physiol. (1996) 496:551–7. doi: 10.1113/jphysiol.1996.sp021706, PMID: 8910237 PMC1160898

[54] Rodrigues P Correia MdeA Wharton L . Effect of menstrual cycle on muscle strength. J Exerc Physiol. (2019) 22:89–97.

[55] Ansdell P Brownstein CG Škarabot J Hicks KM Simoes DCM Thomas K . Menstrual cycle-associated modulations in neuromuscular function and fatigability of the knee extensors in eumenorrheic women. J Appl Physiol. (2019) 126:1701–12. doi: 10.1152/japplphysiol.01041.2018, PMID: 30844334

[56] Romero-Moraleda B Del Coso J Gutierrez-Hellin J RuizMoreno C Grgic J Lara B . The influence of the menstrual cycle on muscle strength and power performance. J Hum Kinet. (2019) 68:123–33. doi: 10.2478/hukin-2019-0061, PMID: 31531138 PMC6724592

[57] Hertel J Williams NI Olmsted-kramer LC Leidy HJ Putukian M . Neuromuscular performance and knee laxity do not change across the menstrual cycle in female athletes. Knee Surg Sports Traumatol Arthrosc. (2006) 14:817–22. doi: 10.1007/s00167-006-0047-4, PMID: 16470385

[58] Otaka M Chen SM Zhu Y Tsai YS Tseng CY Fogt DL . Does ovulation affect performance in tennis players? BMJ Open Sport Exerc Med. (2018) 4:e000305. doi: 10.1136/bmjsem-2017-000305, PMID: 29464104 PMC5812395

[59] Quigley T Greig M . The influence of menstrual cycle phase on isokinetic knee flexor and extensor strength in female soccer players: a pilot study. Res Sports Med. (2025) 33:87–96. doi: 10.1080/15438627.2024.2420085, PMID: 39470599

[60] Dos Santos Andrade M Mascari NC Foster R de JÁrMy di Bella ZI Vancini RL Barbosa de Lira CA . Is muscular strength balance influenced by menstrual cycle in female soccer players? J Sports Med Phys Fitness. (2017) 57:859–64. doi: 10.23736/S0022-4707.16.06290-3, PMID: 27045741

[61] Shahraki SF Minoonejad H Tabrizi YM . Comparison of some intrinsic risk factors of shoulder injury in three phases of menstrual cycle in collegiate female athletes. Phys Ther Sport. (2020) 43:195–203. doi: 10.1016/j.ptsp.2020.02.010, PMID: 32220759

[62] Tasmektepligil MY Agaoglu SA Turkmen L Türkmen M . The motor performance and some physical characteristics of the sportswomen and sedentary lifestyle women during menstrual cycle. Arch Budo. (2010) 6:195–203.

[63] Smith MJ Adams LF Schmidt PJ Rubinow DR Wassermann EM . Effects of ovarian hormones on human cortical excitability. Ann Neurol. (2002) 51:599–603. doi: 10.1002/ana.10180, PMID: 12112106

[64] Lowe DA Baltgalvis KA Greising SM . Mechanisms behind estrogen’s beneficial effect on muscle strength in females. Exerc Sport Sci Rev. (2010) 38:61. doi: 10.1097/JES.0b013e3181d496bc, PMID: 20335737 PMC2873087

[65] Huang G Bhasin S Pencina K Cheng M Jasuja R . Circulating dihydrotestosterone, testosterone, and free testosterone levels and dihydrotestosterone-to-testosterone ratios in healthy women across the menstrual cycle. Fertil Steril. (2022) 118:1150–8. doi: 10.1016/j.fertnstert.2022.09.011, PMID: 36371319

[66] Taylor S Islam RM Bell RJ Hemachandra C Davis SR . Endogenous testosterone concentrations and muscle mass, strength and performance in women, a systematic review of observational studies. Clin Endocrinol. (2023) 98:587–602. doi: 10.1111/cen.14874, PMID: 36585396

[67] Kissow J Jacobsen KJ Gunnarsson TP Jessen S Hostrup M . Effects of follicular and luteal phase-based menstrual cycle resistance training on muscle strength and mass. Sports Med. (2022) 52:2813–9. doi: 10.1007/s40279-022-01679-y, PMID: 35471634

[68] Giacomoni M Bernard T Gavarry O Altare S Falgairette G . Influence of the menstrual cycle phase and menstrual symptoms on maximal anaerobic performance. Med Sci Sports Exerc. (2000) 32:486–92. doi: 10.1097/00005768-200002000-00034, PMID: 10694136

[69] García-pinillos F Bujalance-moreno P Lago-fuentes C Ruiz-Alias SA Domínguez-Azpíroz I Mecías-Calvo M . Effects of the menstrual cycle on jumping, sprinting and force-velocity profiling in resistance-trained women: A preliminary study. Int J Environ Res Public Health. (2021) 18:4830. doi: 10.3390/ijerph18094830, PMID: 33946536 PMC8124250

[70] Kishali NF Kiyici F Burmaoglu G Tas M Paktas Y Ertan F . Some performance parameter changes during menstrual cycle periods of athletes and non-athletes. Ovidius Univ Ann Ser Phys Educ Sport Sci. (2010) 10:46–9.

[71] Smirniotou A Nikolaou C Tsolakis C . The effect of menstrual cycle phases on fencers’ neuromuscular performance. (Poster Session). In 2004 Pre-Olympic Congress: Proceeding: Volume II: Posters: Sport Science through the Ages, Thessaloniki, Greece: Aristotle University Campus. (2004).

[72] Ghazel N Souissi A Chtourou H Aloui G Souissi N . The effect of music on short-term exercise performance during the different menstrual cycle phases in female handball players. Res Sports Med. (2022) 30:50–60. doi: 10.1080/15438627.2020.1860045, PMID: 33291988

[73] Martin D Sale C Cooper S Elliott-Sale KJ . Period prevalence and perceived side effects of hormonal contraceptive use and the menstrual cycle in elite athletes. Int J Sports Physiol Perform. (2018) 13:926–32. doi: 10.1123/ijspp.2017-0330, PMID: 29283683

[74] Bruinvels G Burden R Brown N Richards T Pedlar C . The prevalence and impact of heavy menstrual bleeding among athletes and mass start runners of the 2015 London Marathon. Br J Sports Med. (2016) 50:566–6. doi: 10.1136/bjsports-2015-095505, PMID: 26612843

[75] Coelho AR Cardoso G Brito ME Gomes IN Cascais MJ . The female athlete triad/relative energy deficiency in sports (RED-S). Rev Bras Ginecol Obstet. (2021) 43:395–402. doi: 10.1055/s-0041-1730289, PMID: 34077990 PMC10304901

[76] Esin Ergin AK . Menstrual cycle and sporting performance perceptions of elite volleyball players. Int J Appl Exerc Physiol. (2020) 9:57–64.

[77] Bjacobson BH Lentz W . Perception of physical variables during four phases of the menstrual cycle. Percept Mot Skills. (1998) 87:565–6. doi: 10.2466/pms.1998.87.2.565, PMID: 9842602

[78] Kanellakis S Skoufas E Simitsopoulou E Migdanis A Migdanis I Prelorentzou T . Changes in body weight and body composition during the menstrual cycle. Am J Hum Biol. (2023) 35:e23951. doi: 10.1002/ajhb.23951, PMID: 37395124

[79] Dye L Blundell JE . Menstrual cycle and appetite control: implications for weight regulation. Hum Reprod. (1997) 12:1142–51. doi: 10.1093/humrep/12.6.1142, PMID: 9221991

[80] Stachenfeld NS Dipietro L Kokoszka CA Silva C KeefE D Nadel E . Physiological variability of fluid-regulation hormones in young women. J Appl Physiol. (1999) 86:1092–6. doi: 10.1152/jappl.1999.86.3.1092, PMID: 10066728

[81] Baker FC Siboza F Fuller A . Temperature regulation in women: Effects of the menstrual cycle. Temperature (Austin Tex.). (2020) 7:226–6. doi: 10.1080/23328940.2020.1735927, PMID: 33123618 PMC7575238

[82] Somboonwong J Chutimakul L Sanguanrungsirikul S . Core temperature changes and sprint performance of elite female soccer players after a 15-minute warm-up in a hot-humid environment. J Strength Cond Res. (2015) 29:262–9. doi: 10.1097/JSC.0000000000000235, PMID: 25532432

[83] Tingle C Vora S . Break the Barriers: Girls’ experiences of menstruation in the UK (2018). Plan International UK. Available online at: https://plan-uk.org/act-for-girls/girls-rights-in-the-uk/break-the-barriers-our-menstrual-manifesto (Accessed March 20, 2024).

[84] Findlay RJ Macrae EH Whyte IY Easton C Forrest LJ . How the menstrual cycle and menstruation affect sporting performance: experiences and perceptions of elite female rugby players. Int J Br J Sports Med. (2020) 54:1108–13. doi: 10.1136/bjsports-2019-101486, PMID: 32349965

[85] Becker AJ . It’s not what they do, it’s how they do it: athlete experiences of great coaching. Int J Sports Sci Coa. (2009) 4:93–119. doi: 10.1136/bjsports-2019-101486, PMID: 32349965

[86] Kroshus E Sherman RT Thompson RA Sossin K Austin SB . Gender differences in high school coaches’ Knowledge, attitudes, and communication about the female athlete triad. Eat Disord. (2014) 22:193–208. doi: 10.1080/10640266.2013.874827, PMID: 24456303

[87] Reade I Rodgers W Norman L . The under-representation of women in coaching: A comparison of male and female canadian coaches at low and high levels of coaching. Int J Sports Sci Coa. (2009) 4:505–20. doi: 10.1260/174795409790291439

