# Supplementary figures and images for: Exercise performance at different phases of the menstrual cycle: measurements, differences, and mechanisms - a narrative review
Source: Front Endocrinol (Lausanne). 2025 Dec 16;16:1448686. doi: 10.3389/fendo.2025.1448686 (PMC12747961; doi:10.3389/fendo.2025.1448686)

### Literature Review Process

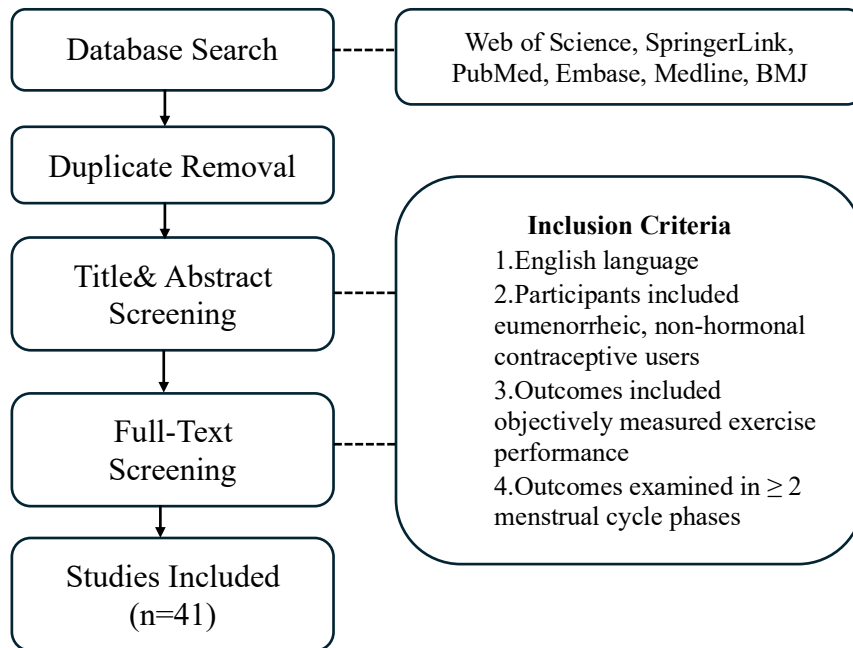

Supplement: Supplementary Figure — Summary of the search and screening methods implemented in this narrative review. [file Image1.pdf]
